# Embryonic transcriptome sequencing of the ocellate spot skate *Okamejei kenojei*

**DOI:** 10.1038/sdata.2018.200

**Published:** 2018-10-08

**Authors:** Chiharu Tanegashima, Osamu Nishimura, Fumio Motone, Kaori Tatsumi, Mitsutaka Kadota, Shigehiro Kuraku

**Affiliations:** 1 Laboratory for Phyloinformatics, RIKEN Center for Biosystems Dynamics Research, 2-2-3 Minatojimaminami-machi, Chuo-ku, Kobe, Hyogo, 650-0047, Japan; 2 Phyloinformatics Unit, RIKEN Center for Life Science Technologies, 2-2-3 Minatojimaminami-machi, Chuo-ku, Kobe, Hyogo, 650-0047, Japan; 3 Graduate School of Science and Technology, Kwansei Gakuin University, Sanda, Hyogo, 669-1337, Japan

**Keywords:** Evolutionary developmental biology, Gene expression analysis, RNA sequencing, Embryology

## Abstract

Chondrichthyans (cartilaginous fishes) exhibit highly variable reproductive styles, categorized as viviparity and oviparity. Among these, species with oviparity provide an enormous potential of molecular experimentation with stable sample supply which does not demand the sacrifices of live mothers. Cartilaginous fishes are divided into two subclasses, chimaeras (Holocephali) and elasmobranchs (Elasmobranchii), and the latter consists of two monophyletic groups, Batoidea (rays, skates and torpedoes) and Selachimorpha (sharks). Here we report transcriptome assemblies of the ocellate spot skate *Okamejei kenojei*, produced by strand-specific RNA-seq of its embryonic tissues. We obtained a total of 325 million illumina short reads from libraries prepared using four different tissue domains and assembled them all together. Our assembly result confirmed the species authenticity and high continuity of contig sequences. Also, assessment of its coverage of pre-selected one-to-one orthologs supported high diversity of transcripts in the assemblies. Our products are expected to provide a basis of comparative molecular studies encompassing other chondrichthyan species with emerging genomic and transcriptomic sequence information.

## Background & Summary

Oviparous (egg-laying) chondrichthyans are distributed into three different selachimorphan (shark) orders, Carcharhiniformes, Heterodontiformes, and Orectolobiformes, and one batoid (skate) order Rajiformes (summarized in^1^). The order Rajiformes consists of four families, Anacanthobatidae, Arhynchobatidae, Gurgesiellidae, and Rajidae. As of February 2018, molecular sequence data for rajiforms concentrate on species in Rajidae, such as the little skate *Leucoraja erinacea*, whose genome is being sequenced^2^, in the head of the list, and it is followed by *Amblyraja radiata* and *Raja clavata*. One of the genera in Rajidae is *Okamejei*, and among more than a dozen of species in the genus *Okamejei*, we focused on the ocellate spot skate *Okamejei kenojei* in the present study. Previously, this species was recognized as *Raja porosa*, *Okamejei porosa*, or *Raja kenojei*. The NCBI RefSeq entry for the whole mitochondrial genome sequence of this species *Okamejei kenojei* (NC_007173.1) was originally registered in GenBank under one of the former species names, *Raja porosa* (AY525783.1)^3^. The habitat of *O. kenojei* is the coasts of Japan, Korea, China, and Taiwan in the Northwest Pacific^4^. While the skate species with the most abundant sequence information, *L. erinacea*, inhabits a small area on the east coast of North America, *O. kenojei* is one of the most promising oviparous skate species available in East Asia for experimentation.

For modern life science studies, genomic and transcriptomic information serves as an indispensable fundamental resource. Especially for a species without abundant molecular sequences, even small-scale transcriptome sequencing with short-read data acquisition can provide a valuable start point, which can be achieved by simple tissue sampling and small financial investment. For efficient transcriptome sequencing data acquisition, a number of technical factors have been considered to optimize sample preparation, sequencing run design, and post-sequencing data analysis. Previously, the authors’ group proposed some of those factors^5^, and in the present study, we extend those factors by incorporating latest reagents for sample preparation and tools for sequence data analysis.

## Methods

### Animal sampling and RNA extraction

**Figure 1:**
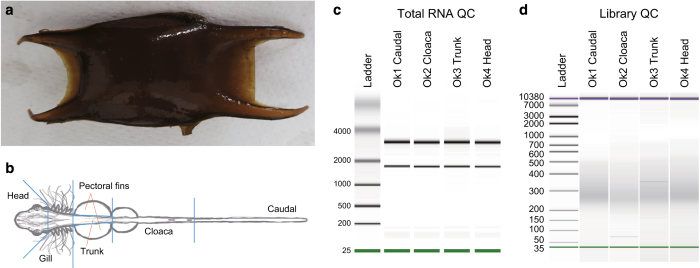
Preparation of the ocellate spot skate RNA-seq library. (**a**) Eggcase. (**b**) Embryo dissection. (**c**) Length distributions of the extracted total RNAs. (**d**) Length distributions of DNA molecules in the prepared RNA-seq libraries. The peak lengths were 261 (Ok1 Caudal), 259 (Ok2 Cloaca), 253 (Ok3 Trunk), and 263 bp (Ok4 Head).

All animal experiments were conducted in accordance with the Guideline of the Institutional Animal Care and Use Committee (IACUC) of RIKEN Kobe Branch (Approval ID: H16-11). An approximately 8 cm-long fertilized egg of the ocellate spot skate, *O. kenojei* (Fig. 1a), was purchased from a commercial marine organism supplier in Minami-ise town in Mie Prefecture, Japan, in February 2016. The embryo contained in the eggcase was 5 cm-long, corresponding morphologically to the stage 31 of typical shark embryos^6,7^ and was dissected into six pieces labelled as head, gill, trunk, pectoral fins, cloaca, and caudal (Fig. 1b). Total RNAs were extracted from the four parts of the embryo (head, trunk, cloaca, and caudal) using TRIzol reagent (Life Technologies) (Fig. 1c).

### Library preparation and sequencing

Using 1 μg of each of the extracted total RNAs, four strand-specific RNA-seq libraries were prepared with KAPA Stranded mRNA-Seq Kit (Kapa Biosystems, cat. No. KK8420) according to its standard protocol unless stated otherwise below. Before the total volume PCR amplification was performed, we performed a preliminary PCR using a 1.5 μl aliquot of 10 μl DNA from the previous step, with KAPA Real-Time Library Amplification Kit (Kapa Biosystems, cat. No. KK2702). This demonstrated that the amplification of the products reached Standard 1 accompanying this kit between three and four PCR cycles, which instructed us to perform the full-volume PCR with three PCR cycles, introducing the minimal amplification (Fig. 1d). The libraries were first sequenced in-house after on-board cluster generation for 127 cycles using 3x HiSeq Rapid SBS Kit v2-HS (50 cycles) (Illumina, cat. No. FC-402-4022) and HiSeq PE Rapid Cluster Kit v2-HS (Illumina, cat. No. PE-402-4002) on a HiSeq 1500 (Illumina) operated by HiSeq Control Software v2.2.58. The output was processed with Illumina RTA v1.18.64 for basecalling and with bcl2fastq v1.8.4 for de-multiplexing. To obtain more reads, we outsourced the sequencing for three of the four above-mentioned libraries on HiSeq 4000. Quality control of the obtained fastq files for individual libraries was performed with FASTQC v0.11.5 (https://www.bioinformatics.babraham.ac.uk/projects/fastqc/).

### Read trimming and assembly

**Figure 2:**
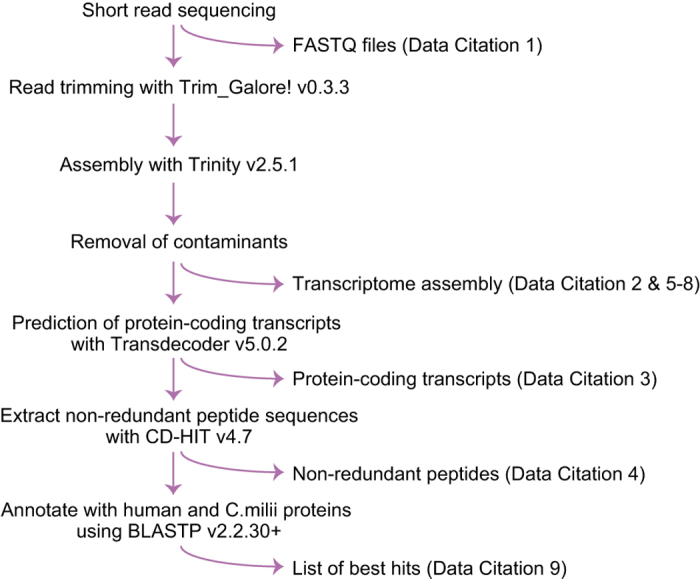
Overview of the post-sequencing work flow.

The obtained sequence reads in the fastq files (Data Citation 1) were processed with the program Trim Galore! v0.3.3 (https://www.bioinformatics.babraham.ac.uk/projects/trim_galore/) with the options ‘--phred33 --stringency 2 --quality 30 --length 25 --paired’. The reads after adaptor trimming were assembled with the program Trinity v2.5.1^8^ with the options ‘--trimmomatic --min_kmer_cov 2 --SS_lib_type RF’. Among the resultant contig sequences, those matching PhiX, mitochondrial DNA, and genome and transcriptome assemblies of the species sequenced in the same HiSeq run in the BLASTN v2.2.30+^9^ results executed with the options ‘-perc_identity 95’ were removed. We designated the resultant sequence set as the redundant nuclear transcriptome assembly (Data Citation 2). In this assembly, protein-coding regions were predicted with the program Transdecoder v5.0.2^10^ following its user documentation, which employed the protein-level similarity to SWISSPROT^11^ and PFAM^12^ by the programs BLASTP v2.2.30+ and Hmmer v3.1b2^13^, respectively (Data Citation 3). The obtained amino acid sequences were processed by CD-HIT v4.7^14^ with its default parameters to reduce redundancy in it (Data Citation 4). Annotation of the putative peptide sequences were performed with similarity searches using BLASTP v2.2.30+ towards RefSeq Protein sequences for the human (GRCh38, 113,620 sequences - as of July 12, 2018) and the *Callorhinchus milii* (NCBI RefSeq, 28,600 sequences - as of July 12, 2018). The whole post-sequencing procedure is outlined in Fig. 2.

### Code availability

No custom computer code was employed in this study.

## Data Records

The approximately 325 million raw sequence reads from four different portions of a *O. kenojei* embryo were released in the NCBI Sequence Read Archive (Table 1 and Data Citation 1). The nuclear transcriptome assembly using all the obtained reads consisted of 1,081,614 sequences (Data Citation 2), which could include spurious intergenic transcripts and alternative splicing variants. Mapping of the reads employed in the assembly to the assembled transcript contig sequences (Data Citation 2) yielded the mapping rates of as high as 90.2 to 93.1%. The nuclear protein-coding transcriptome assembly consisted of 167,783 sequences available both in the nucleotide and amino acid sequences (Data Citation 3). Out of those putative protein-coding sequences, 88,376 (52.7%) were predicted to have complete ORFs with start and stop codons. The amino acid sequence dataset after removing the redundancy consists of 79,083 components (Data Citation 4). Transcriptome assembly was also performed for the individual libraries for caudal, cloaca, trunk, and head samples, which resulted in 498,477, 377,609, 448,394, and 342,765 contigs, respectively (Data Citation 5, 6, 7, 8). These data are available as multifasta files at FigShare. We also provide a table containing best BLASTP hits to the human and *C. milii* RefSeq Peptide sequences as a reference for cross-species annotation (Data Citation 9).

## Technical Validation

### Contiguity of RNA-seq reads

Next, we validated the contiguity of the transcript sequences by short read assembly. For this purpose, we focused on known transcript sequences of *O. kenojei* available at NCBI Nucleotide (https://www.ncbi.nlm.nih.gov/nuccore) that are longer than 1 Kbp and derived from the nuclear genome (with the filter ‘Okamejei kenojei[Organism] AND 1000:1000000[slen] NOT mitochondrial’, accessed on March 15, 2018). With this criterion, we have identified five sequence entries (AB371645.1, AB295474.1, AB195842.1, AB201248.1, and AB201247.1), and for each of those sequences, a transcript contig in our assembly showed no less than 98% similarity (no more than 1% gaps) (Table 2). These high similarities authentificate that the species used for this study was *O. kenojei.*

### Transcript diversity measured by one-to-one ortholog coverage

We also employed a completeness assessment on the web server gVolante^15^ in which the ortholog search pipeline BUSCO^16^ is implemented. This method evaluates the coverage of one-to-one protein-coding orthologs selected in advance. For this purpose, we used the ortholog set CVG^5^ introduced for more accurate assessment for vertebrate sequence sets than performed with other ortholog sets, as well as the Vertebrata ortholog set released together with BUSCO^16^. Although the assessment results showed a slight fluctuation depending on the type of input data (nucleotide or amino acid sequences), out of the 233 components of CVG, our resultant data were shown to always contain at least 218 full-length ortholog sequences (‘complete’) and 232 partial sequences (‘fragmented’) (Table 3). Our assessment with the BUSCO’s Vertebrata ortholog set also produced comparable scores, mostly >92% in percentages. These high scores ascertain the high coverage of protein-coding genes in our resultant transcript sequence data set.

## Additional information

**How to cite this article**: Tanegashima, C. *et al*. Embryonic transcriptome sequencing of the ocellate spot skate *Okamejei kenojei*. *Sci. Data*. 5:180200 doi: 10.1038/sdata.2018.200 (2018).

**Publisher’s note**: Springer Nature remains neutral with regard to jurisdictional claims in published maps and institutional affiliations.

## Supplementary Material



## Figures and Tables

**Table 1:** Sequencing statistics.

**Library ID**	**Tissue**	**Peak of library molecule length distribution (nt)**	**Sequencing configuration**	**# of raw read pairs obtained**	**# of qualified read pairs**	**SRA ID**
Ok1	Caudal	272	HiSeq 1500 paired-end 127nt	81,561,105	80,108,371	SRR6866827
			HiSeq 4000 paired-end 101nt	29,204,559	28,792,861	SRR6866830
Ok2	Cloaca	257	HiSeq 1500 paired-end 127 nt	53,239,929	52,302,602	SRR6866826
Ok3	Trunk	275	HiSeq 1500, paired-end127nt	35,956,038	34,786,361	SRR6866825
			HiSeq 4000 paired-end 101nt	28,423,526	27,887,860	SRR6866829
Ok4	Head	269	HiSeq 1500 paired-end 127nt	59,350,049	58,390,345	SRR6866824
			HiSeq 4000 paired-end 101nt	37,010,620	36,489,494	SRR6866828
All the total RNA samples exhibited the RIN of 10, and all the resultant libraries were amplified with as few as three PCR cycles.						

**Table 2:** Long contig sequence matches to existing database entries.

**Existing NCBI Entry**			**Match in transcript assembly**			
**NCBI ID**	**Gene**	**Length (nt)**	**Transcript ID**	**Length (nt)**	**% Identity**	**% Gap**
AB371645.1	ptx-l	1,416	DN146223_c3_g1_i4	1,077	99	0
AB295474.1	ptx	1,540	DN136073_c1_g3_i2	584	98	0
AB195842.1	SkCOL1A1	4,866	DN146743_c0_g1_i26	3,558	99	0
AB201248.1	COL2A1	1,802	DN128141_c7_g2_i3	4,913	99	0
AB201247.1	COL5/11A1	6,451	DN133476_c9_g3_i7	2,630	98	1

**Table 3:** Sequence datasets produced in this study.

**Dataset**	**Number of sequences**	**Number of core genes** a **CVG (Vertebrata BUSCO)**	**N50 contig length (bp)**	**Data Records**		
		**Only ‘Complete’**	**Including ‘Fragmented’**	**‘Missing’**		
Transcriptome assembly - all libraries	1,081,614	218 (2407)	232 (2524)	1 (62)	992	Data Citation 2
Transcriptome assembly - caudal	498,477	226 (2423)	229 (2496)	4 (90)	1,679	Data Citation 5
Transcriptome assembly - cloaca	377,609	224 (2371)	230 (2476)	3 (110)	1,744	Data Citation 6
Transcriptome assembly -trunk	448,394	225 (2389)	232 (2489)	1 (97)	1,560	Data Citation 7
Transcriptome assembly - head	342,765	221 (2361)	228 (2454)	5 (132)	1,889	Data Citation 8
Protein-coding assembly - all libraries	167,783	218 (2401)	232 (2514)	1 (72)	1,782	Data Citation 3
Non-redundant peptides - all libraries	79,083	219 (2400)	233 (2514)	0 (72)	N/A	Data Citation 4

^a^See the existing literature^16^ for the definitions of ‘complete’, ‘fragmented’ and ‘missing’ in ortholog detection by BUSCO. CVGs consists of 233 orthologs in total, while Vertebrata BUSCO has 2,586 orthologs.
